# Association Between FABP3 and FABP4 Genes with Changes in Milk Composition and Fatty Acid Profiles in the Native Southern Yellow Cattle Breed

**DOI:** 10.3390/vetsci12090893

**Published:** 2025-09-15

**Authors:** Mervan Bayraktar, Serap Göncü, Atalay Ergül, Recep Karaman, Bahri Devrim Özcan, Şerife Ergül, Celile Aylin Oluk, Özgül Anitaş, Ahmet Bayram, Mohammed Baqur S. Al-Shuhaib

**Affiliations:** 1 Department of Animal Science, Faculty of Agriculture, Çukurova University, Adana 01330, Turkey; sgoncu@cu.edu.tr (S.G.); dozcan@cu.edu.tr (B.D.Ö.); ozgulanitas01@gmail.com (Ö.A.); ariahmet74@gmail.com (A.B.); 2 Eastern Mediterranean Agricultural Research Institute Directorate, Adana 01000, Turkey; atalayergul01@gmail.com (A.E.); recep.karaman@tarimorman.gov.tr (R.K.); serife.ergul@tarimorman.gov.tr (Ş.E.); celileaylin.oluk@tarimorman.gov.tr (C.A.O.); 3 Department of Animal Production, College of Agriculture, Al-Qasim Green University, Babylon 51001, Iraq; mohammed79@agre.uoqasim.edu.iq

**Keywords:** native southern yellow, FABP3, FABP4, milk composition, milk fatty acid profile, SNP

## Abstract

Fatty acid binding proteins FABP3 and FABP4 act as intracellular lipid chaperones that influence fatty acid transport and metabolism in mammary tissue, and genetic variation in these genes may affect milk composition. We examined the associations between FABP3 and FABP4 polymorphisms and milk composition and fatty acid profiles in 200 lactating Native Southern Yellow (NSY) cows. DNA from each cow was PCR-amplified and Sanger-sequenced for FABP3 and FABP4; genotypes were tested for their association with milk fatty acid concentrations and standard composition traits using linear models adjusted for relevant covariates. We detected a missense variant in FABP3 (c.3656G > A; p.Val45Met) and an intronic SNP in FABP4 (g.3509T > C). The FABP3 p.Val45Met AA genotype was associated with higher concentrations of butyric, palmitic, oleic, and α-linolenic acids. Cows with the FABP4 TC genotype exhibited elevated levels of myristoleic, γ-linolenic, conjugated linoleic, and arachidic acids, along with increased fat-free dry matter, protein, and lactose. In silico analyses provided mixed evidence for the structural effects of p.Val45Met, molecular docking suggested altered ligand affinity for several fatty acids, and splice site prediction implicated g.3509T > C in possible transcript processing changes. These variants constitute candidate markers for milk fatty acid composition in NSY cattle; replication in independent cohorts and functional validation are recommended to confirm their utility for milk quality improvement.

## Simple Summary

The quality of milk and the types of fats it contains are important for human health and for the dairy industry, and small differences in animals’ genes can change milk composition. We studied 200 Native Southern Yellow cows to see whether natural variations in two genes that help move and store fats in mammary cells—fatty acid binding protein 3 and fatty acid binding protein 4—are linked to differences in milk nutrients and fat composition. We found two genetic differences: one in the fatty acid binding protein 3 gene and one in the fatty acid binding protein 4 gene. Cows with these variants showed higher amounts of some saturated and unsaturated fatty acids and, for one variant, higher levels of protein and lactose in milk. Computer analyses suggested that these changes might affect how the proteins work or how the gene is processed, but the evidence is not yet definitive. These genetic markers are promising candidates for breeding programs aimed at improving milk quality, although further studies in other herds and laboratory tests are needed to confirm their practical value.

## 1. Introduction

Cattle continue to play a central role in global food security and rural livelihoods by providing milk, meat, and other animal products that support human nutrition and household incomes [1]. The recent intensification of dairy production using high-yielding, cosmopolitan breeds has increased output but raised concerns about the loss of locally adapted variation and reduced resilience under marginal environments [2,3]. Indigenous populations frequently harbor alleles that confer adaptation to local feeding regimes, climatic stress, and endemic disease pressures; therefore, the molecular characterization of these genetic resources is essential both for conservation and for the targeted improvement of product quality under low-input management [4,5].

Milk fat composition significantly influences the functionality and nutritional quality of dairy products. The relative proportions of saturated (SFAs), monounsaturated (MUFAs), and polyunsaturated fatty acids (PUFAs) influence the melting point, flavor, oxidative stability, and potential health effects of dairy foods [6,7]. While diet and stage of lactation are significant modulators of milk fatty acid profiles, genetic factors also contribute substantially: heritability estimates for many fatty acid traits are moderate, indicating additive genetic variance that can be exploited by selection [8,9]. Identifying functionally relevant loci that regulate lipid uptake, intracellular transport, and triglyceride assembly, therefore, offers a route to improve milk quality through marker-assisted or genomic approaches.

Fatty acid binding proteins (FABPs) form a family of small cytosolic proteins (≈14–15 kDa) that reversibly bind long-chain fatty acids and related hydrophobic ligands, facilitating their intracellular trafficking and partitioning toward storage, oxidation, or membrane synthesis [10,11]. In the lactating mammary gland, FABPs are involved in channeling fatty acids into the triglyceride synthesis pathway and in regulating lipid droplet formation. Two members, FABP3 (heart-type FABP) and FABP4 (adipocyte-type FABP), have been highlighted in lipid metabolism studies and are expressed in tissues relevant to milk fat synthesis [12,13]. Previous breed-level association studies and the mapping of FABP4 to a QTL-rich region for milk traits further motivated their selection as candidate loci for both association testing and structure–function modeling [14,15]. The bovine FABP3 gene is located on chromosome 2 and comprises four exons and three introns. In contrast, the bovine FABP4 gene, which also consists of four exons and three introns, is located on chromosome 14 within a QTL-rich interval for milk fat percentage and yield.

The FABP3 gene encodes a 132 amino acid protein expressed in cardiac and skeletal muscle and in lactating mammary tissue. Functional assays in bovine mammary epithelial cells show that FABP3 overexpression enhances lipid droplet accumulation upon exposure to oleic, stearic, and palmitic acids, supporting a direct role in cellular fatty acid handling [13,16]. Candidate gene association studies have reported links between FABP3 variants and milk fat traits in several breeds. However, effect direction and significance often vary by population and marker, suggesting population-specific allele effects [14,17]. FABP4 encodes a protein that interacts with PPARγ-mediated lipogenic networks. FABP4 variants and haplotypes have been associated with alterations in medium- and long-chain fatty acid proportions and with production traits in multiple dairy populations [15,18,19,20].

Although FABP3 and FABP4 have been studied in cosmopolitan dairy breeds, their polymorphic diversity and phenotypic consequences in indigenous populations such as the NSY remain underexplored. Characterizing these genes in locally adapted cattle is important because allele frequencies, haplotype backgrounds, and gene–environment interactions may differ substantially from those in intensively selected populations, potentially revealing breed-specific variants that affect milk quality under local feeding and management systems [21,22].

Complementary to classical genotype–phenotype associations, in silico prediction and structural modeling provide tools to infer plausible molecular mechanisms by which sequence variants can alter protein function. Functional predictors (e.g., SIFT, PolyPhen-2, PROVEAN) evaluate the likely impact of amino acid substitutions on protein activity or stability. At the same time, splice site prediction algorithms assess whether intronic variants may affect pre-mRNA processing. Molecular docking and homology modeling further allow for the comparison of ligand binding affinities and interaction geometries between wild-type and mutant proteins, an approach that, when paired with association evidence, can strengthen causal inference about how specific alleles influence phenotypes [23,24,25,26].

Given the central role of FABP3 and FABP4 in fatty acid handling and the limited data available for Turkish indigenous cattle, the present study integrates genetic association and in silico functional analyses in NSY cows. This study aimed to identify single nucleotide polymorphisms in FABP3 and FABP4 in NSY cows, test their associations with milk composition and individual fatty acid concentrations, and assess potential functional consequences using multiple computational prediction tools and molecular docking analyses.

## 2. Materials and Methods

### 2.1. Animal Resources

**Figure 1:**
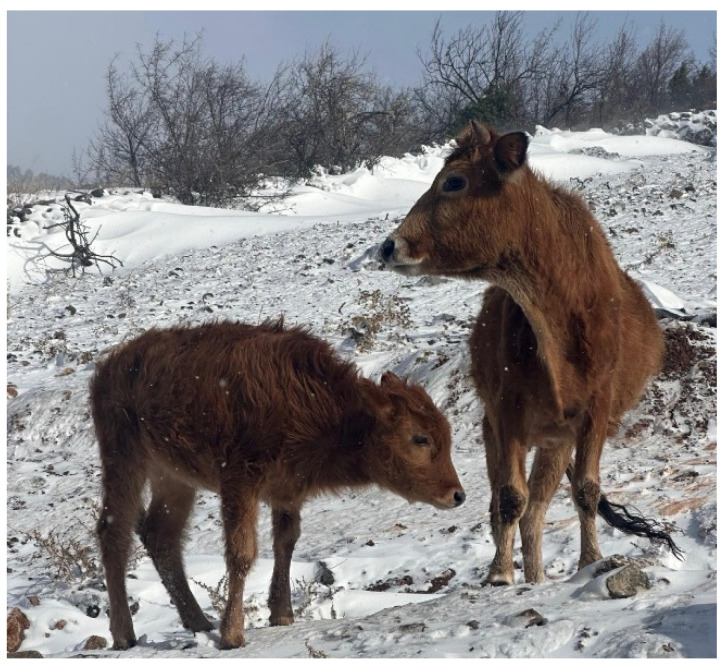
NSY breed.

Ethical approval for all animal procedures was granted by the Çukurova University Local Ethics Council for Animal Experiments (HADYEK; approval no. 2-2-2024). All experimental protocols adhered to the relevant Turkish regulations on animal welfare and experimentation. A total of 200 adult female NSY cattle were sampled from two privately operated farms in Gürümze village, Feke District, Adana Province, Türkiye (Figure 1). Peripheral blood sampling took place in October–November 2024. All sampled animals were actively lactating at the time of sampling and were included according to the following age criterion: only cows aged 4–5 years were enrolled (age verified from farm birth records and by dental eruption patterns). Collecting 200 eligible animals was challenging because herds are small and dispersed in rugged terrain and because local management practices include informal cross-breeding that complicates the identification of unequivocally purebred individuals; we therefore prioritized animals that owners identified as NSY and that displayed breed-typical phenotypes when pedigree records were not available. Breed-typical morphometric and production characteristics reported for the NSY include average female live weights between ~197 and 306 kg, with typical withers heights of ~93–120 cm, lactation lengths of ~158–235 days, and lactation yields ranging approximately 444–794 L per lactation with milk fat values commonly between ~3.0% and 4.1%. Animals were maintained under traditional extensive village husbandry, with seasonal transhumance to surrounding mountain pastures. Each spring (April), animals are moved to higher-elevation rangelands where they graze freely for approximately five months before returning to the village in late autumn/winter (snowfall season). Summer pastures are natural, non-improved rangelands characterized by xerophytic perennial grasses, annual forbs, and scattered shrubs and trees, typical of the Taurus–Amanos mountain fringe. The region’s climate is Mediterranean, with hot, dry summers and wetter, cooler winters. For each sampled cow, we recorded animal ID, age, and parity. At sampling, herd sizes were [two herds totaling 200 cows; distribution by herd: farm A = 98 cows, farm B = 102 cows], and herd management consisted of open grazing with minimal concentrate supplementation during the grazing season and additional supplementation in village rations during the winter months. Peripheral blood samples (10 mL) were collected by jugular venipuncture into EDTA tubes, transported on ice, and stored at −20 °C until DNA extraction was performed. Milk samples for composition and fatty acid analyses were collected during morning milking under hygienic conditions and labeled to ensure direct linkage between milk composition data and the corresponding DNA/molecular data for each animal.

### 2.2. Milk Analyses

Milk samples were collected once during morning milking by hand-stripping each of the four udder quarters into sterile containers. Equal volumes from the four quarters were pooled to produce a single composite sample per cow; two sterile 50 mL Falcon tubes (containing equal aliquots of the composite) were prepared for each animal, immediately chilled on ice, and transported to the laboratory, where they were stored at −20 °C until analysis. Each sample was labeled with a unique animal ID to maintain linkage between milk composition, fatty acid, and DNA data. Milk composition parameters (total solids, non-fat solids, fat, protein, lactose, conductivity, freezing point, and density, as well as free fatty acids and citric acid where applicable) were measured for each composite sample using a Milkana Multi-Test SAC/10 analyzer (GNC Agriculture, Konya, Türkiye) according to the manufacturer’s instructions. The Milkana Multi-Test SAC/10 offers rapid, multi-parameter measurements suitable for both the field and laboratory screening of milk quality. Total lipid extraction for fatty acid analysis was performed according to [27]. Fatty acid methyl esters (FAMEs) were prepared from the extracted lipid following a standard methanolysis procedure [28]. In brief, 25 mg of extracted oil was added to 4 mL of 2 M KOH in methanol and 10 mL of n-heptane, vortexed for 1 min, and then centrifuged at 3500 rpm for 10 min. The upper n-heptane layer, containing FAMEs, was transferred to GC vials for analysis. All reagent volumes and steps were followed according to the cited protocols to ensure efficient transesterification. FAME separation and quantification were carried out using gas chromatography on an Agilent 7890A GC system (Agilent Technologies) equipped with a flame ionization detector (FID). An Agilent J&W DB-WAX capillary column (30 m × 0.25 mm i.d., 0.25 µm film thickness) was used. The operating conditions were as follows: injector temperature 250 °C; detector (FID) temperature 260 °C; helium as the carrier gas at ~1 mL/min; split ratio 1:50. All GC injections were performed in technical duplicate, and the fatty acid composition is reported as mean ± standard deviation (percentage of total peak area).

### 2.3. DNA Isolation

Up to 10 mL of blood samples was drawn from the coccygeal vein of 200 cows, placed in EDTA tubes containing gel, and gently inverted to prevent hemolysis and clotting. The withdrawn blood samples were placed in an ice box and transported to the Animal Science Laboratory at Çukurova University, where they were stored at −20 °C until processing. Genomic DNA was extracted from whole blood using the GeneJET Genomic DNA Purification Kit (Thermo Fisher Scientific, Waltham, MA, USA; Cat. #K0722) according to the manufacturer’s instructions, with a brief protocol as described here. In short, 200–500 µL of whole blood was mixed with lysis solution and 20 µL of proteinase K, then incubated at 56 °C for 10–30 min to ensure complete cell lysis and protein digestion. Then, an equal volume of absolute ethanol was added to promote DNA binding. The lysate was applied to the silica spin column and centrifuged; the column was washed twice with the provided wash buffer to remove contaminants, and DNA was eluted in 50–100 µL of the supplied elution buffer (or nuclease-free water) by a brief incubation at room temperature followed by centrifugation. To monitor DNA quality and quantity, sample purity (A260/A280) and concentration were measured using a NanoDrop spectrophotometer (Thermo Fisher Scientific, Waltham, MA, USA; Cat. #ND-2000), and DNA integrity for a subset of samples was checked on a 0.8% agarose gel. Samples of genomic DNA were stored at −20 °C until they were used for downstream PCR amplification and sequencing. DNA samples with A260/A280 ratios of ~1.8–2.0 and intact high-molecular weight bands on agarose gel were used for subsequent genotyping.

### 2.4. PCR Design and Amplification

Using Primer3web (v.4.1.0) [29], we designed gene-specific primer pairs to amplify regions of FABP3 and FABP4. FABP3 primers (targeting exon two and flanking intronic sequence; ~600 bp; GenBank NC_037329.1) were as follows: F 5′-CGTTAAGTTTCTCATGTCTCATG-3′ and R 5′-GTCCAGTCCCACAGGCAACAGGT-3′. FABP4 primers (spanning intron 2, exon three, and part of intron 3; ≈720 bp; GenBank NC_037341.1) were as follows: F 5′-TAATAAAATTGTCCTTATTACTT-3′ and R 5′-TGAGAGGGATAAGAAAATACTGC-3′. PCRs were performed in 25 µL reactions containing 1× PCR buffer, 2 mM MgCl_2_ (2 µL of 25 mM stock), 200 µM of each dNTP, 0.5 µM of each primer, 1 U Taq DNA polymerase, and ~50 ng genomic DNA. Thermal cycling consisted of an initial denaturation at 95 °C for 5 min, followed by 35 cycles of 95 °C for 30 s, annealing (FABP3: 60 °C for 45 s; FABP4: 54 °C for 60 s), and 72 °C for 60 s, with a final extension at 72 °C for 5 min. PCR products were verified using agarose gel electrophoresis and subsequently sequenced using the Sanger method.

### 2.5. DNA Sequencing and Interpretation

Before sending samples to sequencing laboratories, PCR products were resolved by 2% (*w*/*v*) agarose gel electrophoresis, and FABP3 and FABP4 bands of the expected sizes were purified using a silica membrane column-based cleanup kit (EURx, Gdańsk, Poland, Cat# E3520). Subsequently, the samples were prepared according to the standard guidelines provided by the sequencing facility (Macrogen Inc., Seoul, Republic of Korea). Subsequently, Sanger’s sequencing was performed in the forward direction for all analyzed samples using the respective primers. The DNA sequences of the sequencing files were then aligned with the reference sequences of the targeted genes using BioEdit software (Version 7.0.0., 2004. Available online: https://bioedit.software.informer.com/ (accessed on 10 June 2025)) [30]. For alignment and variant calling, we used the Bos taurus RefSeq transcripts NM_174313.2 (FABP3) and NM_174314.2 (FABP4), and all variant positions reported in the manuscript are given with respect to the Bos taurus ARS-UCD1.2 reference genome assembly (GCF_002263795.1).

The identified SNPs were confirmed on the raw data (ab.format) of the electropherograms using the SnapGene viewer tool, version 4.0.4. (Dotmatics, available at snapgene.com). Upon inspection of the chromatogram peaks for each detected SNP, the variants were verified as genuine and not attributable to technical artifacts [31]. By reviewing genetic variant tables for the bovine FABP3 and FABP4 genes in the Ensembl genome browser release 114 (https://www.ensembl.org/index.html) (accessed on 12 August 2025) [32], the novelty of each detected SNP is confirmed. To validate the genetic annotations, each identified sequence was submitted to the NCBI BankIt portal [33], and a unique GenBank accession number was assigned to each entry.

### 2.6. Statistical Analyses

Allele and genotype frequencies, tests for Hardy–Weinberg’s equilibrium (HWE), genetic diversity indices, and other population–genetic parameters were calculated using Popgen32 (version 1.32). Chi-square (χ^2^) tests assessed deviations of observed genotype distributions from HWE expectations. Associations between genotype and phenotypic traits were evaluated via a General Linear Model (GLM), specified as

*Y_ijks_* = *μ* + *α_i_* + *C_k_* + *D_s_* + *ε_ijks_*

where

*Y_ijks_* is the trait value of the *j*th individual with genotype *i*, on farm *k*, age *s*;
*μ* is the overall mean;
*α_i_* is the fixed effect of genotype;
*C_k_* is the fixed effect of the farm;
*D_s_* is the fixed effect of age;
*ε_ijks_* is the random error term.

Least square means (LSmeans) and their standard errors were estimated from the fitted GLM. Pairwise comparisons of LSmeans between genotypes were performed using the Tukey honestly significant difference test (Tukey). Tukey’s test was used to adjust pairwise contrasts and thereby control the family-wise type I error rate across genotype comparisons.

### 2.7. Predicting the Impact of the Missense SNP on FABP3 Structure and Function

Since the three-dimensional structure of the bovine FABP3 protein had been previously deposited in a crystallized form [34], the structure (PDB ID: 1BWY) was retrieved from the RCSB Protein Data Bank (https://www.rcsb.org/ (accessed on 1 July 2025)). The downloaded crystallized structure was used as a template to predict the potential effects of the identified missense SNP on the protein’s structure/function and stability. To evaluate the structural and functional impact of the identified missense SNP on FABP3, seven in silico prediction tools were employed to provide a cumulative assessment of the overall effect of the variant on the target protein. The FABP3 amino acid sequences were submitted as FASTA sequences to these tools to assess their consequences on protein structure and function. SIFT (Sorting Intolerant From Tolerant) can predict whether an amino acid substitution affects protein function based on sequence homology and the physical properties of amino acids [35]. PolyPhen-2 (Polymorphism Phenotyping v2) assesses the possible impact of an amino acid substitution on the structure and function of a human protein using physical and comparative considerations. This tool combines sequence conservation and structural features to classify variants as benign, possibly damaging, or probably damaging [36]. PhD-SNP (Predictor of Human Deleterious Single Nucleotide Polymorphisms) is used to employ protein sequence information and evolutionary conservation to evaluate the effect of the variants [37]. The SNPs&Go tool (https://snps-and-go.biocomp.unibo.it/snps-and-go/ (accessed on 1 August 2025)) is used to integrate functional annotations from protein sequences with evolutionary conservation to predict the potential deleterious effects of amino acid variants [38]. SNAP (Screening for Non-Acceptable Polymorphisms) is a neural network-based tool designed to consider both sequence and structural features to classify variants as either neutral or deleterious to protein function [39]. Meta-SNP is a consensus predictor that integrates outputs from multiple in silico methods to generate a more reliable prediction of SNP effects (https://snps.biofold.org/meta-snp/ (accessed on 1 July 2025)). PredictSNP is another consensus method that aggregates predictions from various computational tools to classify SNPs as neutral or deleterious [40]. Each of these tools relies on a distinct algorithm to assess whether a missense SNP is neutral or harmful.

### 2.8. Predicting the Impact of the Missense SNP on FABP3 Stability

After evaluating the impact of each SNP on protein structure and function, its effect on protein stability was cumulatively assessed using an additional set of seven tools. The PDB structure of the FABP3 protein (1BWY) was used as a template for this prediction. The mCSM (mutation Cutoff Scanning Matrix) tool quantifies the impact of the SNP on protein stability by employing graph-based structural signatures [41]. Likewise, the assessment of a given SNP’s effect on protein stability, as well as SDM (Site-Directed Mutator), is used. The tool employs environmental substitution tables that are derived from known protein structures to assess the stabilizing or destabilizing effects on the protein [42]. Additionally, DUET is utilized to combine the prediction of both mCSM and SDM into a consensus framework to improve accuracy in estimating thermodynamic changes upon mutation [43]. The DDGun 0.0.2 tool is used to provide a quantitative measurement of the SNP’s effect on protein stability. This can be performed by predicting the change in Gibbs free energy (ΔΔG) associated with the missense SNP [44]. Mupro predictor is also employed due to its capacity to use machine learning approaches to estimate stability changes [45]. To calculate the effect of an SNP on folding stability, PremPS (Prediction of Protein Mutant Stability) is used. This software is a web server tool that predicts the impact of an SNP on protein stability by combining structure-based energy functions and statistical potentials [46]. The last stability prediction tool used is I-Mutant2. This tool is a widely used server to predict the direction and magnitude of the effect of an SNP on the stability changes in the affected protein [47]. Subsequently, the expected impact of the SNP on FABP3 was graphically illustrated, and the SNP was mapped onto the 3D structure of both the wild-type and mutant proteins using the mutagenesis script in PyMOL [48].

### 2.9. Molecular Docking of FABP3 with Fatty Acids

To further assess the impact of the detected missense SNP on the interaction of FABP3 with its substrates, molecular dockings were performed between the wild-type FABP3 and its mutant form with three common fatty acids. Three fatty acids known to bind with FABP3 with high affinity were selected in these dockings. These fatty acids were palmitic acid (PubChem ID 985), linoleic acid (PubChem ID 5280934), and oleic acid (PubChem ID 445639). After retrieving these compounds from the PubChem server in 3D SDF format (https://pubchem.ncbi.nlm.nih.gov/ (accessed on 5 June 2025)), they were docked with both the wild-type and mutant FABP3 using the SwissDock tool (https://www.swissdock.ch/ (accessed on 5 June 2025)). This tool is a type of freely available, web-based molecular docking software that enables the simulation of molecular interactions between ligands and protein targets. It is characterized by its potential to automate ligand and target preparation and compute possible binding modes by exploring the whole protein surface or specified regions of interactions [49]. Using the recommended algorithms of the Autodock-vina docking method [50], two dockings were performed for each selected ligand, one with the wild-type FABP3 and one with the mutant FABP3. For all dockings conducted, a 20 Å cubic docking box was centered at coordinates (X = 0, Y = −1, Z = −4) around the FABP3 protein. The default search exhaustiveness value of 4 was used to allow the ligand to explore possible orientations and conformations within the cavity, thereby generating predicted binding poses and scores. The pose with the best docking score was selected, and PyMOL was employed to compare the interaction efficiency between the wild-type and mutant FABP3 proteins. Furthermore, protein–ligand interactions were annotated using Discovery Studio software [51].

### 2.10. Predicting the Impact of FABP4 Intron SNP

To evaluate the possible effect of the identified intron SNP on the contribution of the FABP4 transcript in the splicing patterns, the ESEfinder software, release 3.0, was employed [52]. This piece of software is an online server used to predict the potential impact of intronic variants on altering gene expression. This prediction can be generated by using exon splicing enhancers. These enhancers are sequence motifs that promote exon recognition potentials during the splicing of pre-mRNA [53]. Based on the default setting of the server accessed in July 2025, the splicing patterns of the wild-type FABP4 and its alternative form were compared, and the scores of Serine/Arginine-rich (SRp) components were used to evaluate the differences between them. The predicted values of the ESEfinder tool were displayed as bars of varying lengths, indicating the positions of motifs that may contribute to alterations in splicing patterns [54].

## 3. Results

### 3.1. Genotype and Allele Frequencies

**Figure 2:**
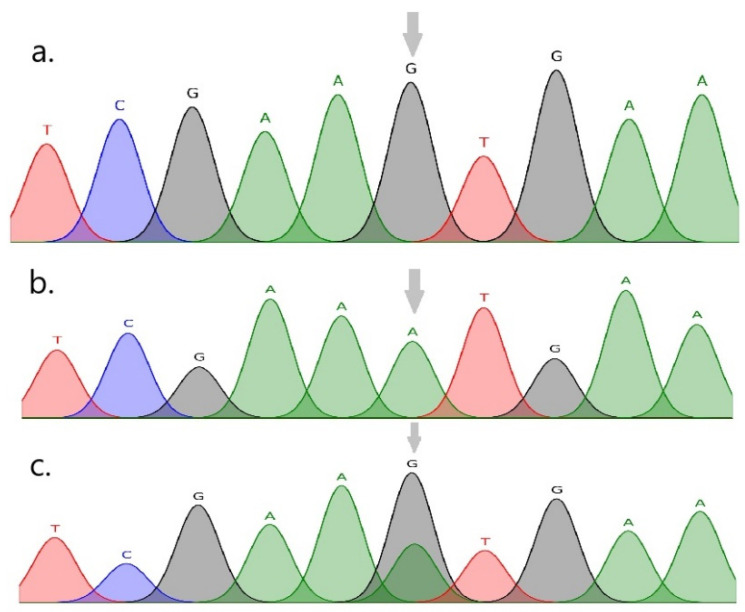
FABP3 (Chr 2): c.3656G > A → p.Val45Met (exon 2; reference NC_037329.1). Sequencing electropherogram showing the c.3656G > A substitution: (**a**) GG genotype, (**b**) AA genotype, (**c**) GA genotype.

**Figure 3:**
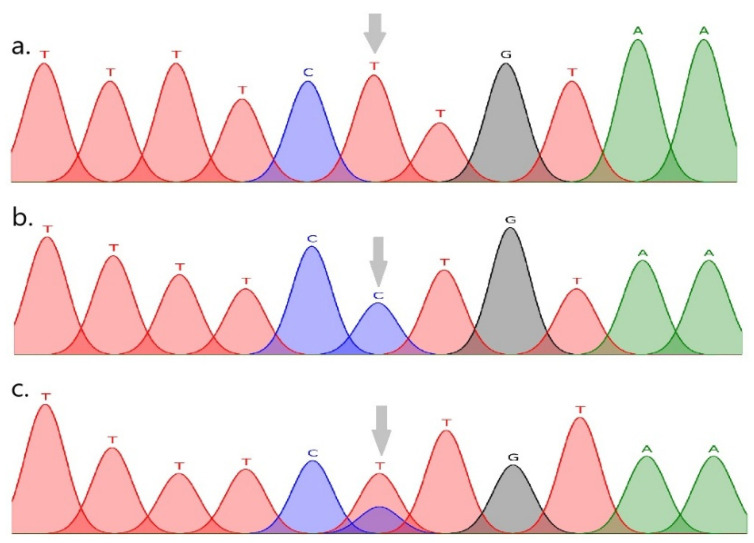
FABP4 (Chr 14): g.3509T > C (intron 2; reference NC_037341.1). Sequencing electropherogram showing the g.3509T > C substitution: (**a**) TT genotype, (**b**) CC genotype, (**c**) TC genotype.

Sequencing and genotyping analyses of the FABP3 and FABP4 genes in the indigenous NSY cattle breed revealed one SNP in each gene. The multiple nucleotide alignment of the identified FABP3 sequences with the corresponding reference sequence (GenBank accession NC_037329.1) showed a single substitution of G → A at nucleotide position 273 of the 600 bp PCR amplicon. This variant corresponds to c.3656G > A in the FABP3 coding sequence and is located in exon 2 (Figure 2). The FABP3 variant is a missense (nonsynonymous) substitution, c.3656G > A (p.Val45Met), i.e., the replacement of valine (Val, V) by methionine (Met, M) at residue 45 of the bovine FABP3 protein. The novelty of the identified p.Val45Met variant was confirmed by an inspection of the bovine FABP3 variant table (ENSBTAG00000016819) in Ensembl release 114. The alignment of the FABP4 sequences with the reference (GenBank accession NC_037341.1) revealed a single T → C substitution at nucleotide position 319 of the 720 bp PCR amplicon, corresponding to g.3509T > C in the genomic context. This variant is located in intron 2 of FABP4 (Figure 3). The novelty of the identified intronic variant was confirmed by an inspection of the bovine FABP4 variant table (ENSBTAG00000037526) in Ensembl release 114.

The genotype and allele frequencies are presented in Table 1. For FABP3, the GG, GA, and AA genotypes were observed at frequencies of 62.5%, 25.0%, and 12.5%, respectively, corresponding to G and A allele frequencies of 75.0% and 25.0%, respectively. A chi-square test for HWE indicated a significant deviation (*p* < 0.05), and the expected heterozygosity (*H*e) was calculated as 0.38, indicating a moderate level of genetic diversity at this locus. For FABP4, the TT, TC, and CC genotypes occurred at frequencies of 65.0%, 10.0%, and 25.0%, respectively, yielding T and C allele frequencies of 70.0% and 30.0%, respectively. The FABP4 locus likewise deviated significantly from HWE (*p* < 0.05). With an *H*e value of 0.42, the polymorphic FABP4 locus has also reflected another moderate level of genetic variability.

To validate our genetic annotations for the analyzed FABP3 and FABP4 genes, each observed genotype has been deposited in NCBI and has received a unique GenBank accession number. For FABP3, the GenBanks PX121467, PX121468, and PX121469 were obtained to represent the GG, GA, and AA genotypes, respectively. For FABP4, the GenBanks PX121470, PX121471, and PX121472 were obtained to characterize the TT, TC, and CC genotypes, respectively.

### 3.2. Association Between the FABP3 Polymorphism and Milk Fatty Acid Profile

The relationship between the FABP3 c.3656G > A polymorphism and milk fatty acid composition is summarized in Table 2. Four fatty acids differed significantly among genotypes (*p* < 0.05). Butyric acid (C4:0) concentrations were 2.05 ± 0.05% in GG, 2.09 ± 0.04% in GA, and 3.09 ± 0.06% in AA animals, with the AA genotype showing significantly higher levels than the other two groups (*p* = 0.037). Palmitic acid (C16:0) averaged 35.26 ± 0.97% for GG, 37.43 ± 0.73% for GA, and 40.36 ± 1.01% for AA, with AA again exhibiting the highest mean (*p* = 0.014). Oleic acid (C18:1 cis-9) levels were 16.82 ± 0.77%, 17.28 ± 0.58%, and 18.89 ± 0.80% in the GG, GA, and AA genotypes, respectively, and differed significantly among groups (*p* = 0.043). For α-linolenic acid (C18:3 cis-9, cis-12, cis-15), the AA genotype displayed the highest concentration (1.17 ± 0.16%), which was significantly greater than the other genotypes (GG: 0.70 ± 0.16%, GA: 0.81 ± 0.12%; *p* = 0.010), although the difference between GG and GA was not significant. No significant genotype-dependent differences were observed for the remaining fatty acids (C8:0, C10:0, C12:0, C14:0, C14:1, C15:0, C16:1, C17:0, C18:0, C18:2, C17:1, γ-linolenic acid, conjugated linoleic acid, and arachidic acid) (*p* > 0.05) (Table 2).

### 3.3. Association Between FABP4 Polymorphism and Milk Fatty Acid Composition

Milk fatty acid profiles were compared across FABP4 g.3509T > C genotype groups (TT, TC, and CC), and the mean ± SE values along with significance levels are presented in Table 3. Myristoleic acid (C14:1 cis-9) concentrations differed significantly among genotypes (*p* = 0.031), with TT animals exhibiting 1.49 ± 0.07, TC animals 2.28 ± 0.05, and CC animals 1.30 ± 0.06. Gamma-linolenic acid (C18:3) also showed a significant genotype effect (*p* = 0.017), being lowest in TT cows (1.36 ± 0.04), highest in TC cows (2.37 ± 0.08), and intermediate in CC cows (1.44 ± 0.11). The proportion of conjugated linoleic acid (cis-9, trans-11 CLA) varied significantly by genotype (*p* = 0.027), with values of 3.44 ± 0.20 in the TT group, 4.80 ± 0.25 in the TC group, and 2.31 ± 0.27 in the CC group. Arachidic acid (C20:0) concentrations likewise differed among genotypes (*p* = 0.041), with TT, TC, and CC cows recording 3.58 ± 0.30, 5.74 ± 0.26, and 4.83 ± 0.38, respectively. No statistically significant differences were observed among genotypes for the remaining fatty acids (C4:0, C8:0, C10:0, C12:0, C14:0, C15:0, C16:0, C16:1, C17:0, C18:0, C18:1 cis-9, C18:2, C18:3 cis-9, cis-12, cis-15, and C17:1) (*p* > 0.05) (Table 3).

### 3.4. Association Between FABP3 Polymorphism and Milk Composition

The effects of the FABP3 c.3656G > A polymorphism on general milk composition are summarized in Table 4. Among the measured components of fat content, solids not fat (SNF), density, protein concentration, freezing point, milk temperature, lactose, electrical conductivity, and pH, only milk fat percentage differed significantly by genotype (*p* = 0.039). Cows with GG, GA, and AA genotypes exhibited mean fat contents of 1.16 ± 0.49%, 1.67 ± 0.38% and 2.37 ± 0.53%, respectively, with the AA genotype showing the highest fat proportion. No significant effects of genotypes were detected on SNF, density, protein, freezing point, temperature, lactose, conductivity, or pH (*p* > 0.05) (Table 4).

### 3.5. Association Between FABP4 Polymorphism and Milk Composition

Milk compositional traits were evaluated with respect to the FABP4 g.3509T > C polymorphism (Table 5). No significant effect of genotype was observed for milk fat percentage (*p* = 0.437) or for the majority of physicochemical traits (freezing point, temperature, electrical conductivity, and pH; *p* > 0.05). In contrast, milk density differed significantly among genotypes (*p* = 0.029). Solids not fat (SNF) did not show a genotype effect (*p* = 0.512). Both protein and lactose concentrations were significantly associated with genotype (protein: *p* = 0.021; lactose: *p* = 0.010). Detailed least square means and standard errors for each genotype group are provided in Table 5.

### 3.6. Impact of the Missense p.Val45Met on the FABP3 Protein

**Figure 4:**
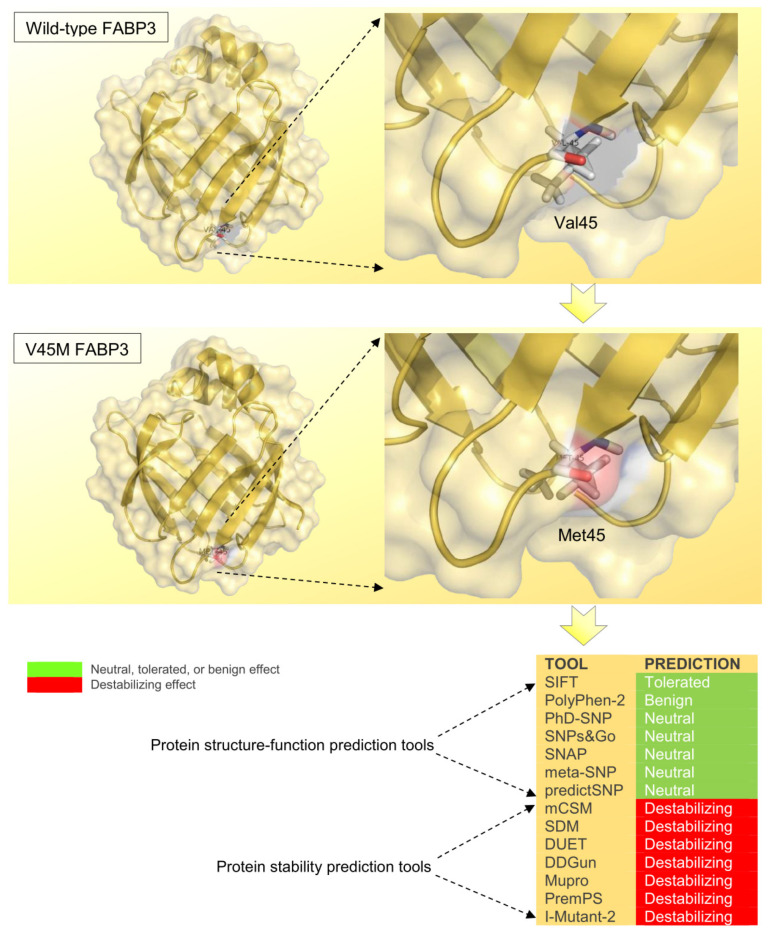
Prediction of the effect of the identified p.Val45Met SNP on the structure-function, and stability of the FABP4 protein. The missense SNP is shown as sticks within the cartoon structure, surrounded by a transparent surface of the FABP3 protein.

To examine the structural context of the identified p.Val45Met (Val→Met) substitution, we mapped the residue onto the FABP3 model. We found that it localizes at the junction between a β-sheet and an adjacent loop, a position that can affect local folding and ligand access. We applied two complementary groups of in silico predictors: (i) function/impact tools (e.g., SIFT, PolyPhen-2, PhD-SNP, PredictSNP) to assess likely functional consequences, (ii) stability predictors (e.g., mCSM, SDM, DUET, I-Mutant2) to estimate effects on protein stability. Function-oriented predictors collectively indicated a neutral effect of p.Val45Met on FABP3 activity, whereas stability predictors consistently suggested a destabilizing effect (Figure 4). To help reconcile this difference, we performed molecular docking, which indicated altered ligand interactions in the mutant protein, with increased predicted affinity for several tested fatty acid ligands. Taken together, these results suggest that although the substitution may not abolish protein function, it could alter protein stability and ligand binding dynamics. This hypothesis requires experimental validation (e.g., binding assays, thermal shift assays, or cellular functional assays).

### 3.7. Molecular Docking Outputs

**Figure 5:**
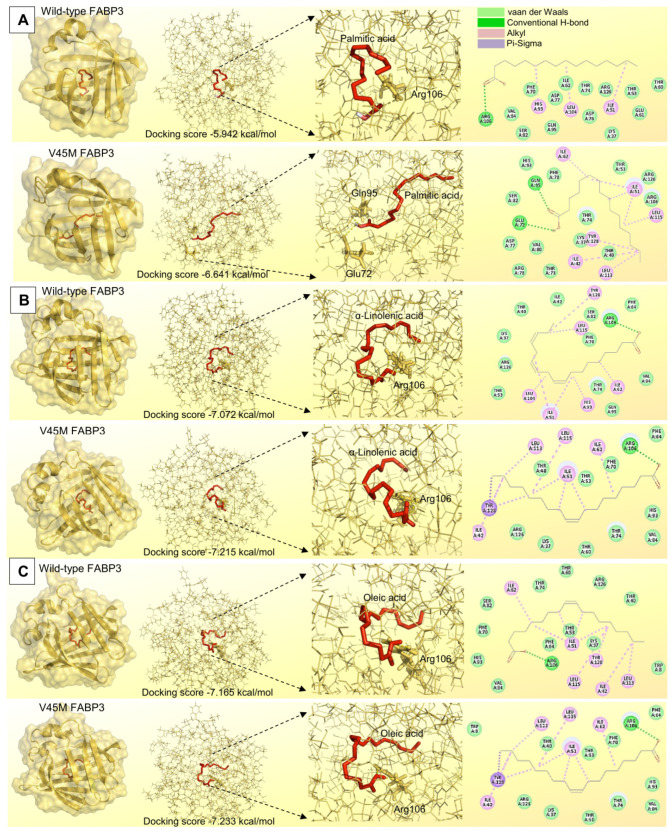
Comparative molecular docking interactions between wild-type FABP3 and p.Val45Met FABP3 regarding their binding with palmitic acid, α-linolenic acid, and oleic acid in panels (**A**–**C**), respectively.

To evaluate whether the p.Val45Met substitution affects ligand binding, we performed molecular docking of three representative fatty acid ligands (palmitic, α-linolenic, and oleic acids) to wild-type and p.Val45Met FABP3 models. The docking results indicate modest but consistent increases in the predicted binding affinity of all three ligands to the p.Val45Met model relative to wild type, accompanied by changes in the expected interaction patterns. In particular, palmitic and α-linolenic acids showed a larger increase in the number of predicted stabilizing contacts in the mutant model. In contrast, oleic acid exhibited only minor changes in interaction geometry but a slight rise in predicted affinity. These observations suggest that the p.Val45Met substitution may alter ligand binding dynamics without abolishing binding capability; detailed docking scores and interaction maps are provided in Figure 5.

### 3.8. The Impact of Intronic g.3509T > C SNP on FABP4 Transcripts

**Figure 6:**
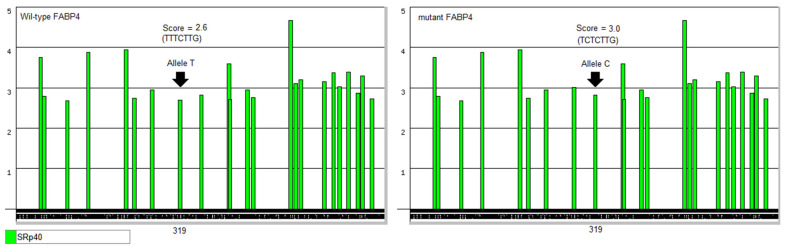
Predicting the effect of the identified g.3509T > C SNP on changing the splicing pattern within the affected intron 2 of the FABP4 gene. The number 319 refers to the position of the detected intronic SNP within the PCR amplicon.

The results from ESEfinder for the identified SNP (g.3509T > C) in the FABP4 gene indicate a change in the splicing regulatory landscape, specifically affecting exonic splicing enhancer (ESE) motifs recognized by splicing factors such as SRp40. In the wild-type allele with T at position 319 of the PCR amplicon, the ESE scores of 2.6 show a specific binding affinity for SRp40. This score is related to the SRp40 motif TTTCTTG. This type of binding may indicate a normal recognition of splicing enhancer elements that contribute to the proper inclusion of exons during mRNA processing. The mutant allele C at the same position caused an alteration in the SRp40 motif to TCTCTTG, which was accompanied by an increase in SRp40 binding, as indicated by a score of 3 (Figure 6). This score suggests that the identified intronic SNP may enhance or create a more favorable ESE motif for this particular splicing factor.

## 4. Discussion

In this study, the genetic polymorphisms in the FABP3 and FABP4 genes have been analyzed in indigenous NSY. The analysis of both genes can be crucial for understanding the possible mechanisms underlying the interactions of FABP3 and FABP4 gene products and their influence on milk fat synthesis and composition. While the FABP3 gene showed a c.3656G > A SNP with a missense impact on the FABP3 protein, the FABP4 gene exhibited a g.3509T > C SNP with an intronic effect on the FABP4 transcripts. Due to the differences in the behavior of each identified SNP, this study detected the different consequences that each SNP has on milk fat and composition. Collectively, our association results and in silico analyses support the study objective by identifying candidate functional variations (p.Val45Met in FABP3 and g.3509T > C in FABP4) that correlate with differences in milk fatty acid profiles and composition, thereby providing testable hypotheses about the molecular mechanisms underlying these phenotypes.

Both identified polymorphic loci within the FABP3 and FABP4 genes have shown significant deviations from HWE in the analyzed NSY population. The possible reason for this inconsistency with HWE is related to the potential influences of non-random mating or selection acting on this population. Though the reported allele frequencies in the analyzed NSY population are broadly comparable to those observed in several *Bos taurus* dairy populations, they continue to reflect apparent breed-specific heterogeneity. While the observed minor allele frequencies (MAFs) were 0.25 for c.3656G > A and 0.30 for g.3509T > C in NSY, the exon 3 SNP of the FABP3 gene reported MAFs of 0.15 in Holstein Friesian cattle [55]. While a Polish Holstein herd showed an MAF value of about 0.30, Jersey cows exhibited a lower MAF value of only 0.11 in the polymorphic locus within FABP4 [14,56]. These differences may be reflected in the effect of breed differences on allele distributions in genes involved in lipid metabolism. Notably, the history of cattle populations and the objectives of selection strategies may influence the variability of FABP3 and FABP4 across different dairy cattle breeds [15,20,57].

Several mechanistic and population–genetic processes may underlie why the associations we observe in NSY cattle differ from those reported in Holstein and other cosmopolitan breeds. Firstly, the distribution of alleles and local patterns of linkage disequilibrium (LD) vary substantially among breeds; consequently, a marker or SNP that is in strong LD with a causative variant in one breed may not tag the same functional variation in another [58,59]. Secondly, divergent selection histories and breeding goals (e.g., intensive selection for milk yield in Holsteins versus adaptation and dual-purpose selection in NSY) can change allele frequencies and functional allele effects across populations [15]. Thirdly, breed-specific differences in mammary gland physiology and the expression of lipid handling pathways (including FABP3/FABP4 and PPAR/SREBP networks) can modulate how genetic variation translates to phenotypes [13,60]. Fourthly, strong genotype × environment (G × E) interactions are reported for milk traits: differences in diet, pasture composition, climate, and management between studies (for example, extensive mountain grazing in our study versus intensive feeding in many Holstein studies) may alter the phenotypic effects of the identical genotypes [61]. Finally, methodological differences, including sample size, parity, and lactation stage control, and whether analyses account for population structure, affect the power to detect additive versus dominance effects and may explain some inconsistent reports [14,62]. Together, these considerations motivate the replication of our findings in larger, multi-breed samples and targeted functional assays to determine whether the detected FABP3 p.Val45Met and FABP4 intronic variants have conserved mechanistic effects across breeds.

Concerning the FABP3 c.3656G > A SNP, this study observed a significant association between this SNP and both milk fat percentage and the concentrations of multiple individual fatty acids. Cows with the minor homozygous AA genotype exhibited higher concentrations of butyric acid (C4:0), palmitic acid (C16:0), oleic acid (C18:1 cis-9), and α-linolenic acid (C18:3) in milk fat than cows with GG and GA genotypes.

The associations we report for the FABP3 gene with milk composition and fatty acid profiles are concordant with multiple prior studies in cattle that link variation at this gene to fat-related milk traits. The identified FABP3 SNP in Jersey cows has been significantly associated with both milk fat and protein percentages [56]. Similarly, Yadav and Mukherjee [17] reported that FABP3 genotypes in Karan Fries cows were related to superior lactation performance. Meanwhile, Yadav et al. [63] found that a heterozygous genotype at an intron-2 FABP3 SNP was favorable for 305-day milk and fat yields in Sahiwal cattle.

While our study found a significant association of the FABP3 c.3656G > A (p.Val45Met) variant with milk fat-related fatty acids, Nafikov et al. [62] reported no significant relationships between FABP3 haplotypes and milk fatty acid composition in Holstein cows; their aggregated haplotypes showed no apparent influence on either the saturated or unsaturated fractions. While several factors can explain the lack of significance reported in Holstein cows, our findings in NSY indicate that the FABP3 locus can exert detectable effects on milk fatty acid composition and related milk composition traits (e.g., fat percentage, fat-free dry matter, protein, and lactose).

Notably, the milk fat percentage in the cows with the AA genotype has been shown to be roughly double that of cows with the GG and GA genotypes. These patterns suggest that the A allele may confer a generalized enhancement of milk fat synthesis or secretion within the udder. Consistent with the broad roles played by FABP3 in lipid metabolism [12], the increased concentrations of these fatty acids may imply that cows with the AA genotype preferentially handle and incorporate long-chain unsaturated fatty acids compared with cows with the other genotypes. The possible mechanism for this finding is attributed to the potential ability of the A allele to modulate the efficiency of FABP3 interaction with fatty acids. Given the importance of FABP3 in facilitating the intracellular transport of long-chain fatty acids to counteract oxidative catabolism [13], any amino acid substitution may affect its function. However, it is not feasible to speculate on the mode of action of this suggestion without employing computational tools to predict the possible mechanism behind this alteration.

A broad spectrum of in silico tools is available to assess the effect of the missense SNP on the protein’s biological activity in various domestic animals [64,65,66]. This availability is attributed to the straightforward effect of the amino acid substitution on the 3D structure and its consequent biological functions. Additionally, the availability of computational tools has provided a considerable opportunity to avoid the possibility of mistaken or biased predictions that can occur when a low number of in silico tools are employed [67]. This information has been supported by reports indicating that at least four in silico tools should be used to gain a sufficient understanding of an SNP’s actual impact on the protein of interest [31,68,69]. For this reason, a total of 14 in silico tools have been used to assess the extent of the detected p.Val45Met SNP effect on the FABP3 protein. Hence, the possibility that the identified p.Val45Met SNP modifies conformation, FA binding affinity, and substrate specificity has been assessed using various in silico tools. The reduced binding strength of FABP3 with fatty acids may decrease their transportation potential, thereby decreasing their relative abundance in milk [13].

By contrast, a variant that increases FABP3 binding efficiency to long-chain fatty acids may enhance intracellular fatty acid binding and trafficking, thereby increasing the availability of substrates for triacylglyceride synthesis in mammary epithelial cells; for this reason, we used additional computational algorithms to assess whether the identified p.Val45Met substitution alters the FABP3 ligand binding capacity [10].

The substitution of valine with methionine can alter the local packing environment within the FAP3 protein. This is due to the longer, more flexible side chain of methionine that may introduce slight steric changes that affect the precise geometry of the protein core. These steric changes may reduce the local rigidity that valine would normally impart. Due to the critical positioning of the Val45 in the junction between the β-sheet and the loop, its substitution with methionine would potentially lead to mild destabilization in this junction. This suggestion has been supported by the critical importance of the β-sheet–loop interface for protein function and molecular activity [70].

The substitution of valine by methionine at position 45 is likely to perturb the local packing environment because methionine’s longer, more flexible side chain can introduce steric strain and alter hydrophobic repacking within the protein core [70,71]. These steric and conformational differences can reduce the local rigidity usually imparted by a β-branched residue such as valine and thereby increase side chain mobility and local backbone flexibility, with potential consequences for thermodynamic stability and functional dynamics [71,72]. Given that Val45 is located at the junction between a β-sheet and an adjacent loop, even modest increases in side chain volume or flexibility are likely to disturb the precise geometry of the sheet–loop interface and to produce a mild destabilization of this junction; the importance of β-sheet–loop packing for protein integrity and activity has been emphasized in structural studies of FABP family proteins and related β-sheet systems [70,73].

Structure and function prediction tools have generally rated this SNP as neutral, indicating that the change did not disrupt the protein’s general activity. Meanwhile, all of the employed protein stability prediction tools predict that the p.Val45Met mutation has a destabilizing effect on the FABP3 protein. This suggests that while the mutation might not directly impair the protein’s function, it is likely to make the protein less stable. In other words, the effect of this SNP can be accomplished by performing a particular degree of unfolding of the FABP3 protein under physiological conditions. Given the neutral effects predicted by all structure–function tools and destabilizing effects predicted by all protein stability tools, it has been found that this substitution is more likely to influence the thermodynamic stability of the protein rather than its intrinsic biochemical function. Molecular docking showed that though the position at which the amino acid substitution has occurred did not interact directly with the fatty acids tested, this alteration has indirectly affected the interaction of the FABP3 with fatty acids. The reason for this effect may be attributed to the critical positioning of the Val45 residue within the 3D structure of FABP3, where the β-sheet region interacts with the loop region. Given the importance of the interface in which the Val45 residue resides, any amino acid substitution in this region may affect the folding of FABP3, thereby altering its mode of interaction with substrates [74].

The p.Val45Met variant is predicted to be neutral by structure–function classifiers but is consistently scored as destabilizing by protein stability predictors, suggesting a greater effect on thermodynamic stability [47,75]. Because Val45 lies at a β-sheet–loop interface that contributes to local packing and ligand access in FABP3, even a modest destabilization at this position can alter local folding and indirectly perturb fatty acid interactions without directly abolishing catalytic or binding residues [73,74].

Due to the significant association detected in this study between the p.Val45Met SNP and palmitic acid, α-linolenic acid, and oleic acid, they were selected to act as substrates for FABP3 in the conducted molecular docking reactions. Although pyruvic acid concentration showed a significant elevation in cows with the AA genotype compared with other cows, it was not selected for molecular docking with FABP3. This is attributed to the chemical properties of pyruvic acid, which lacks the long hydrophobic chain required for high-affinity interaction with FABP3 [76].

The three fatty acids (palmitic, α-linolenic, and oleic) identified by association were chosen for docking because FABP3 preferentially binds long-chain saturated and unsaturated fatty acids [77,78] and has been shown experimentally to bind palmitate and oleate with high affinity [76]. Pyruvic acid, despite its association with genotype, was excluded from docking because it is a small, highly polar three-carbon α-keto acid that lacks the long hydrophobic alkyl chain required to occupy FABP3’s hydrophobic cavity with high affinity [79,80].

Both wild-type FABP3 and p.Val45Met FABP3 have been compared in terms of their binding capacities with palmitic acid, α-linolenic acid, and oleic acid. All docking reactions showed the superiority of the altered FABP3 in binding with these fatty acids compared with the wild-type FABP3. This superiority has been expressed as a higher affinity of binding potentials, which can be explained by the participation of a greater number of interacting bonds or amino acid residues between the altered p.Val45Met FABP3 and fatty acids. Due to the higher affinity exhibited by the altered p.Val45Met FABP3, it is rational to connect this higher potential for molecular interaction with the higher concentrations of these three fatty acids in the milk of cows with the minor homozygous AA genotype.

Structural mapping places Val45 at the interface between a β-sheet and an adjacent loop that contributes to the entrance and shape of the canonical fatty acid binding cavity in FABP3. The substitution of valine by methionine introduces a longer, more flexible, and more polarizable side chain into this junction, which can modulate local packing and backbone geometry. Our in silico analyses suggest two complementary effects that together increase the predicted ligand affinity for the p.Val45Met model. First, the subtle rearrangement of the β-sheet–loop interface widens and/or reshapes the ligand entry pathway and internal cavity, enabling fatty acid chains to adopt conformations that allow more extensive hydrophobic (alkyl) contacts with additional residues. Second, the conformational change propagates to nearby side chains (for example, Gln95 and Glu72 in our models), repositioning polar groups to form new stabilizing contacts (including hydrogen bonding or electrostatic contacts) that were less favored in the wild-type model. These combined increases in hydrophobic surface burial and in the number of stabilizing contacts are consistent with the more negative docking scores and the larger number of predicted interactions observed for palmitic, α-linolenic, and oleic acids in the mutant model. Notably, stability predictors also indicate a modest destabilizing effect of p.Val45Met, implying an increased local flexibility that may enhance conformational sampling and thereby increase the probability of higher-affinity ligand poses without abolishing binding. Although this structural scenario is coherent with our docking results, it remains a model-based hypothesis and requires empirical validation.

Regarding the FABP4 SNP, the intronic g.3509T > C SNP has been shown to have varying degrees of significant association with several milk parameters. This intronic SNP displayed a distinct association pattern characterized by heterozygote advantage. Cows with the TC genotype exhibited substantial increases in several milk fatty acids and multiple non-fat compositional traits compared with both TT and CC homozygotes. This observation has been manifested in cows with the TC genotype, as they exhibited higher concentrations of myristoleic acid, γ-linolenic acid, conjugated linoleic acid, and arachidic acid compared with cows with other genotypes. In parallel, cows with the heterozygous TC genotype also showed higher protein, lactose, and SNF percentages than those with either homozygote class. In contrast to the observed heterozygote advantage in this study, more straightforward additive or recessive effects for FABP4 polymorphisms have been reported in other studies in cattle. This can be observed in Polish Holstein Friesians, where the minor allele at an FABP4 Val110Met SNP has a deleterious effect on fat content compared with wild-type homozygotes [14]. Consistent with the reported minor allele in fat, the minor allele of the FABP4 gene was associated with a higher milk density in Turkish Holsteins [81].

Intronic variants frequently act by altering splicing regulatory elements or isoform balance, rather than by changing the coding sequence. Therefore, an intronic FABP4 change can plausibly modify transcript abundance or splice–isoform ratios, with downstream effects on nutrient partitioning and milk protein synthesis, rather than on whole milk fat percentage [82,83]. Local LD and breed-specific LD patterns may also cause an intronic marker to tag a nearby causal locus [58]. Finally, breed differences in mammary lipid and protein metabolism and the sensitivity of fat% versus protein to short-term environmental variation can make protein or SNF changes easier to detect than modest genetic effects on fat% in single time-point field samples [13,14,20,60,62].

When contrasting our results with prior reports from Holstein and other cosmopolitan breeds, two points stand out. First, the direction and magnitude of genotype–phenotype associations at FABP loci are not uniform across breeds, reflecting differences in allele frequency, LD context, selection history, and management [14,15,62]. Second, the heterozygote advantage observed here for the FABP4 g.3509T > C (TC) genotype associated with higher concentrations of several health-promoting fatty acids (e.g., CLA, γ-linolenic) and elevated protein/SNF in milk suggests a distinct functional effect that may be particularly relevant in locally adapted, extensively managed populations. From a translational perspective, alleles that confer improved milk composition while supporting resilience under low-input and heat-exposed production systems could be valuable for marker-assisted selection (MAS) in thermotolerant breeds. However, we stress that this potential is hypothesis-generating: before considering MAS or genomic selection implementation, the following steps are required (i) the replication of the FABP4 associations in independent, larger cohorts including heat-stressed animals and multiple herds; (ii) the explicit evaluation of genotype × environment interactions (for example, comparing effects under pasture-based, heat-exposed versus intensive, cooled systems) to determine whether the beneficial fatty acid and compositional effects persist under thermal stress; (iii) the assessment of correlated effects on production, fertility, and animal welfare (e.g., milk yield, body condition, rectal temperature, and respiration rate) to ensure no adverse trade-offs; (iv) functional validation (e.g., splicing and expression assays, and phenotyping under controlled heat challenges) to confirm causal mechanisms. If these criteria are met, the FABP4-TC haplotype (or linked causal variants) could be incorporated into genomic prediction models or targeted MAS programs tailored for thermotolerant and low-input breeding objectives, prioritizing both product quality and adaptation.

In contrast to the missense SNP of FABP3, which showed a direct effect on milk fat percentage, the intronic SNP of FABP4 appears to modulate milk composition. This can be attributed to the predominant prevalence of FABP4 in adipose tissue and lactating mammary glands, where it contributes to systemic energy partitioning and lipid metabolism in cattle [84]. The intronic SNP at this gene is presumably influencing mRNA processing by altering the splicing mechanism. The altered FABP4 transcripts may therefore produce genotype-dependent differences in lactation and fatty acid handling. In contrast to the wide range of computational tools that describe the effects of FABP3 missense SNPs, the in silico tools that are available to explain the impact of this FABP4 intronic SNP are limited due to their restriction on the putative role of the intronic SNP to change the splicing pattern. Nevertheless, the conducted ESEfinder analysis performed on the FABP4 intronic variant g.3509T > C indicated that this intronic SNP altered the strength of the ESE motifs. This alteration in the splicing mechanism can modulate spliceosome recruitment at the adjacent exon–intron boundary [85]. Furthermore, it can influence the splicing pattern by potentially enhancing the recruitment of SRp40, which may lead to changes in the efficiency or regulation of exon recognition. Consequently, this SNP could result in differential splicing outcomes of the FABP4 pre-mRNA, such as exon inclusion or skipping, which may impact the structure and function of the resultant protein. Such changes in splicing patterns can affect gene expression and protein isoform diversity. For this reason, it can be stated that the g.3509T > C SNP appears to modify splicing regulatory elements and may have functional consequences by altering the normal splicing behavior of the FABP4 gene in cattle.

While this study identifies candidate functional variants in FABP3 and FABP4 and links them to variations in milk composition and fatty acid profiles in NSY cattle, several limitations should be acknowledged. Firstly, the moderate sample size and uneven genotype counts for some loci limit the statistical power for the fine decomposition of additive versus dominance effects and for the precise estimation of allele substitution effects. Secondly, pedigree or genome-wide relationship data were not available, preventing mixed-model approaches that would better control for relatedness and population structure. Finally, the in silico predictions and docking results reported here are hypothesis-generating and require experimental validation (e.g., splicing/expression assays, ligand binding or thermal shift assays, and cell-based functional studies) to confirm the mechanistic effects. In future work, we plan to increase the cohort size, incorporate genomic/pedigree information, perform allelic substitution and mixed-model analyses, and undertake targeted wet-lab experiments to validate and extend the present findings.

Our findings underscore the contribution of FABP3 and FABP4 to the genetic architecture of milk composition, corroborating earlier reports in cattle while providing added mechanistic resolution. While other comparable studies have reported a significant association between the FAABP3 and FABP4 genes and various milk compositions and fatty acids [14,15,56], our study has provided putative mechanisms for these associations. This mechanism is suggested in cows with the AA genotype, in which the altered p.Val45Met FABP3 binds with palmitic acid, α-linolenic acid, and oleic acid with greater affinity compared with the wild-type FABP3. This higher affinity of binding has been related to the significantly elevated concentrations of these fatty acids in the cows with the AA genotype. This higher affinity of binding may enhance the uptake and intracellular transport of these fatty acids, potentially leading to increased concentrations of these fatty acids in milk. While FABP3 exerted a direct effect on fatty acid metabolism, FABP4 exerted its effect through one intronic SNP. The in silico tool utilized revealed that this SNP alters the splicing pattern of the FABP4 transcript, thereby affecting its expression level. This suggested post-transcriptional effect of FABP4 may explain the significant association of FABP4 with milk composition and fatty acid profile. However, further studies using more wet-lab experiments are required to validate these findings.

## 5. Conclusions

The objectives of this study, to identify single-nucleotide polymorphisms in FABP3 and FABP4 in NSY cattle and to evaluate their associations with milk composition and fatty acid profiles, were achieved. We detected a missense variant in FABP3 (c.3656G > A; p.Val45Met) and an intronic variant in FABP4 (g.3509T > C), and both loci were significantly associated with specific milk constituents and fatty acids in the sampled cohort. These results provide evidence that variation at FABP3 and FABP4 contributes to phenotypic differences in milk fatty acid composition in this indigenous population. Complementary in silico analyses and molecular docking generate plausible mechanistic hypotheses (p.Val45Met may affect FABP3 stability and ligand binding; g.3509T > C may influence FABP4 splicing) that merit targeted experimental follow-up. While the findings are encouraging, we acknowledge the constraints that limit broad extrapolation: single-time-point sampling, a moderate cohort size, and uneven genotype counts for some classes, as well as the absence of pedigree/genomic relationship data. Accordingly, we recommend replicating this study in larger and geographically diverse populations, incorporating pedigree or genomic data with mixed-model analyses to estimate allele substitution effects robustly, and conducting functional assays (e.g., splicing/expression tests for FABP4; binding and stability assays for FABP3) to confirm causality. If validated, these loci could become useful candidates for marker-assisted or genomic selection strategies tailored to local breeding objectives.

## Figures and Tables

**Table 1:** Genotype and allele frequencies at the FABP3 and FABP4 loci.

Gene Locus	Genotype Frequency	Allele Frequency	χ^2^ (HWE)	*H*e
FABP3	GG (0.625) GA (0.25) AA (0.125)	G (0.75) A (0.25)	*p* < 0.05	0.38
FABP4	TT (0.65) TC (0.10) CC (0.25)	T (0.70) C (0.30)	*p* < 0.05	0.42

χ^2^, chi-square; HWE, Hardy–Weinberg’s equilibrium; *H*e, expected heterozygosity.

**Table 2:** Association between the FABP3 c.3656G > A polymorphism and milk fatty acid composition.

Traits	Genotype			*p*-Value
	GG	GA	AA	
Butyric acid (C4:0)	2.05 ± 0.05 ^b^	2.09 ± 0.04 ^b^	3.09 ± 0.06 ^a^	0.037 *
Caprylic acid (C8:0)	1.32 ± 0.05	1.37 ± 0.04	2.37 ± 0.05	0.102
Capric acid (C10:0)	3.51 ± 0.19	3.65 ± 0.14	4.62 ± 0.19	0.347
Lauric acid (C12:0)	4.42 ± 0.29	4.44 ± 0.22	5.42 ± 0.30	0.098
Myristic acid (C14:0)	13.59 ± 0.32	13.53 ± 0.24	15.03 ± 0.33	0.301
Myristoleic acid (C14:1 cis-9)	1.38 ± 0.05	1.34 ± 0.04	2.34 ± 0.05	0.087
Pentadecanoic acid (C15:0)	0.25 ± 0.02	0.27 ± 0.02	0.58 ± 0.02	0.101
Palmitic acid (C16:0)	35.26 ± 0.97 ^ab^	37.43 ± 0.73 ^b^	40.36 ± 1.01 ^a^	0.014 *
Palmitoleic acid (C16:1)	2.60 ± 0.28	2.33 ± 0.21	3.06 ± 0.29	0.412
Stearic acid (C18:0)	6.72 ± 0.61	5.96 ± 0.46	9.10 ± 0.63	0.236
Oleic acid (C18:1 cis-9)	16.82 ± 0.77 ^ab^	17.28 ± 0.58 ^b^	18.89 ± 0.80 ^a^	0.043 *
Linoleic acid (C18:2 cis-9, cis-12)	1.69 ± 0.09	1.70 ± 0.07	2.52 ± 0.10	0.199
α-Linolenic acid (C18:3 cis-9, cis-12, cis-15)	0.70 ± 0.16 ^a^	0.81 ± 0.12 ^a^	1.17 ± 0.16 ^b^	0.010 *
Heptadecenoic acid (C17:1)	2.15 ± 0.06	2.13 ± 0.05	3.11 ± 0.06	0.328
γ-Linolenic acid (C18:3)	1.39 ± 0.06	1.43 ± 0.05	2.35 ± 0.07	0.157
Conjugated linoleic acid (cis-9, trans-11 CLA)	3.55 ± 0.16	3.36 ± 0.12	4.62 ± 0.16	0.083
Arachidic acid (C20:0)	4.10 ± 0.26	4.25 ± 0.19	5.79 ± 0.27	0.595

* (*p* < 0.05) significant. ^a^ Mean labeled a is significantly different from means labeled b; ^b^ mean labeled b is substantially different from means labeled a; ^ab^ mean labeled ab is not significantly different from either a or b (overlaps both).

**Table 3:** Association between the FABP4 g.3509T > C polymorphism and milk fatty acid composition.

Traits	Genotype			*p*-Value
	TT	TC	CC	
Butyric acid (C4:0)	2.04 ± 0.05	3.15 ± 0.04	2.05 ± 0.06	0.412
Caprylic acid (C8:0)	1.33 ± 0.05	2.39 ± 0.04	1.32 ± 0.05	0.369
Capric acid (C10:0)	3.63 ± 0.18	4.64 ± 0.15	3.51 ± 0.21	0.148
Lauric acid (C12:0)	4.52 ± 0.29	5.43 ± 0.23	4.32 ± 0.32	0.091
Myristic acid (C14:0)	14.13 ± 0.31	14.67 ± 0.25	12.35 ± 0.35	0.741
Myristoleic acid (C14:1 cis 9)	1.49 ± 0.07 ^a^	2.28 ± 0.05 ^b^	1.30 ± 0.06 ^ab^	0.031 *
Pentadecanoic acid (C15:0)	0.20 ± 0.04	0.84 ± 0.05	0.26 ± 0.03	0.137
Palmitic acid (C16:0)	37.55 ± 0.96	39.69 ± 0.78	38.81 ± 1.08	0.274
Palmitoleic acid (C16:1)	1.81 ± 0.30	3.53 ± 0.24	2.65 ± 0.31	0.630
Stearic acid (C18:0)	8.04 ± 0.60	9.37 ± 0.49	7.37 ± 0.67	0.111
Oleic acid (C18:1 cis 9)	17.69 ± 0.76	18.96 ± 0.62	17.74 ± 0.85	0.179
Linoleic acid (C18:2 cis 9, cis 12)	1.71 ± 0.010	2.47 ± 0.08	1.73 ± 0.12	0.320
α Linolenic acid (C18:3 cis 9, cis 12, cis 15)	0.43 ± 0.19	0.75 ± 0.15	0.35 ± 0.18	0.204
Heptadecenoic acid (C17:1)	2.09 ± 0.08	3.19 ± 0.06	2.22 ± 0.07	0.444
γ Linolenic acid (C18:3)	1.36 ± 0.04 ^ab^	2.37 ± 0.08 ^a^	1.44 ± 0.11 ^b^	0.017 *
Conjugated linoleic acid (cis 9, trans 11 CLA)	3.44 ± 0.20 ^b^	4.80 ± 0.25 ^a^	2.31 ± 0.27 ^ab^	0.027 *
Arachidic acid (C20:0)	3.58 ± 0.30 ^ab^	5.74 ± 0.26 ^a^	4.83 ± 0.38 ^b^	0.041 *

* (*p* < 0.05) significant. ^a^ Mean labeled a is significantly different from means labeled b; ^b^ mean labeled b is substantially different from means labeled a; ^ab^ mean labeled ab is not significantly different from either a or b (overlaps both).

**Table 4:** Association between the FABP3 c.3656G > A polymorphism and milk composition.

Traits	Genotype			*p*-Value
	GG	GA	AA	
Fat (%)	1.16 ± 0.49 ^ab^	1.67 ± 0.38 ^b^	2.37 ± 0.53 ^a^	0.039 *
Solids not fat (SNF, %)	8.13 ± 0.32	8.54 ± 0.25	9.57 ± 0.35	0.512
Density (D, °T)	28.91 ± 1.25	30.11 ± 0.97	32.07 ± 1.34	0.368
Protein (%)	2.04 ± 0.12	2.20 ± 0.09	3.50 ± 0.13	0.147
Freezing point (FP, °C)	53.23 ± 2.39	56.36 ± 1.85	60.05 ± 2.58	0.249
Temperature (°C)	19.07 ± 0.63	19.30 ± 0.50	21.02 ± 0.70	0.364
Lactose (%)	4.77 ± 0.66	4.22 ± 0.53	5.87 ± 0.74	0.307
Electrical conductivity (mS/cm)	4.42 ± 0.26	4.07 ± 0.19	5.14 ± 0.28	0.097
pH	6.21 ± 0.39	6.85 ± 0.30	7.47 ± 0.41	0.389

* (*p* < 0.05) significant. ^a^ Mean labeled a is significantly different from means labeled b; ^b^ mean labeled b is substantially different from means labeled a; ^ab^ mean labeled ab is not significantly different from either a or b (overlaps both).

**Table 5:** Association between the FABP4 g.3509T > C polymorphism and milk composition.

Traits	Genotype			*p*-Value
	TT	TC	CC	
Fat (%)	1.99 ± 0.49	3.66 ± 0.44	2.06 ± 0.60	0.437
Solids not fat (SNF, %)	30.45 ± 1.23	32.98 ± 1.11	27.67 ± 1.54	0.512
Density (D, °T)	6.40 ± 0.41 ^ab^	8.304 ± 0.40 ^a^	7.48 ± 0.57 ^b^	0.029 *
Protein (%)	2.36 ± 0.12 ^b^	3.19 ± 0.11 ^a^	2.11 ± 0.15 ^b^	0.021 *
Freezing point (FP, °C)	56.55 ± 2.37	59.16 ± 2.14	53.93 ± 2.95	0.745
Temperature (°C)	18.53 ± 0.63	21.15 ± 0.57	18.73 ± 0.78	0.224
Lactose (%)	4.02 ± 0.67	5.02 ± 0.63	3.77 ± 0.84	0.010
Electrical conductivity (mS/cm)	4.25 ± 0.26	5.36 ± 0.24	4.04 ± 0.35	0.393
pH	5.07 ± 0.38	7.68 ± 0.37	6.47 ± 0.47	0.136

* (*p* < 0.05) significant. ^a^ Mean labeled a is significantly different from means labeled b; ^b^ mean labeled b is substantially different from means labeled a; ^ab^ mean labeled ab is not significantly different from either a or b (overlaps both).

## Data Availability

The datasets used and/or analyzed during the current study are available from the corresponding author on reasonable request due to privacy and ethical restrictions.
